# Prevalence of intimate partner violence in patients presenting with traumatic injuries to a Guyanese emergency department

**DOI:** 10.1186/1865-1380-5-23

**Published:** 2012-05-29

**Authors:** Kendra P Parekh, Stephan Russ, David A Amsalem, Navindranauth Rambaran, Shannon Langston, Seth W Wright

**Affiliations:** 1 Department of Emergency Medicine, Vanderbilt University Medical Center, Nashville, TN, USA; 2 Vanderbilt University School of Medicine, Nashville, TN, USA; 3 Georgetown Public Hospital Corporation, Georgetown, Guyana; 4 Department of Emergency Medicine, Vanderbilt University Medical Center, 1313 21st Avenue S., 703 Oxford House, Nashville, TN, 37232-4700, USA

**Keywords:** Intimate partner violence, Spouse abuse, Women’s health, Guyana, Trauma

## Abstract

**Background:**

Intimate partner violence (IPV) occurs throughout the world, and has both short- term and long- term negative health effects. Little is know about the prevalence of IPV in patients presenting to Emergency Departments (EDs) in the developing world. This information is needed to help delineate the scope of the problem and shape effective interventions to combat IPV. The purpose of this study was to determine the prevalence of intimate partner violence in adult patients with acute traumatic injuries presenting to an ED in Georgetown, Guyana.

**Methods:**

Retrospective descriptive analysis of a prospectively collected ED quality assurance database. Patients 18 years or older who presented with a traumatic injury and answered the question “Was the injury inflicted by a domestic partner?” were included in the analysis.

**Results:**

Overall, 38 of 475 (8%) patients admitted to having injuries inflicted by a domestic partner. Thirty- one (81.6%) patients disclosing IPV were female and 7 (18.4%) were male. The self- reported prevalence of IPV in females presenting with traumatic injuries was 16% compared to 2% for males (RR 6.4; 95% CI 2.9-14.3). IPV was the cause of 31 of the 67 (46.3%) women presenting with assaults.

**Conclusions:**

IPV is thought to be a serious problem in Guyana, and this study confirms a high prevalence (16%) of IPV in women presenting with traumatic injuries to the Georgetown Public Hospital Corporation ED. This is likely a significant underestimate of the true prevalence.

## Background

Intimate partner violence (IPV) is prevalent in all societies of the world [1]. In addition to short- term health consequences, IPV results in long- term health problems, and has broad economic and social implications [2-4]. Public health approaches seek to provide early, accurate diagnosis to reduce morbidity and mortality. The healthcare system in general and the ED in particular is important in this regard as it is an opportunity for IPV victims to obtain not only needed medical care, but also entry into the social service, mental health, and judicial systems [5,6]. In the US, female ED patients are receptive to being asked about IPV, and up to 64% of police- identified IPV victims presented to an ED at least once compared to only 21% of age- matched controls [7,8]. Furthermore, when IPV victims were identified in the ED, 35% of victims contacted the community resources provided within 3 months [9].

Guyana is a developing country located on the northern coast of South America. While located on the South American mainland, Guyana is an English- speaking country, and is culturally and economically part of the Caribbean community. The most prevalent form of interpersonal violence in Guyana is IPV, and it is estimated that two- thirds of all Guyanese women will experience IPV during their lifetime [4,10,11].

Unfortunately, many healthcare workers in both developed and developing countries lack the awareness and training to properly identify and respond to the varied health manifestations of IPV [12-14]. A necessary step in the process of educating healthcare workers is documentation of the extent of the problem. Georgetown Public Hospital Corporation (GPHC) in Georgetown, Guyana, is the largest hospital in the country and the main referral center. Thus, the purpose of this study was to determine the prevalence of IPV in adult patients presenting with traumatic injuries to the GPHC ED.

## Methods

This study took place in the ED of Georgetown Public Hospital Corporation, located in Georgetown, Guyana. GPHC is the main teaching hospital for the country of Guyana and serves as the only tertiary care medical center for Guyana. GPHC is also the trauma center for the country, although no formal trauma center designations exist in Guyana. The ED at GPHC has an estimated annual volume of 75,000 patients. At the time of the study the ED was staffed with residents and general medical officers. An emergency medicine residency program began at GPHC in October 2010, but was not in existence at the time of this study.

During a 2- week period in July 2010, registration personnel at the GPHC ED prospectively collected patient information as part of a detailed quality assurance database. This quality assurance project was conducted by the hospital administration for several purposes, including determination of patient volume, volume trends, transfer and referral patterns, left without being seen rates, resource utilization, chief complaint information, and treatment and disposition patterns. These questions were not always asked in private, and friends or family members were potentially present at the time of the interview.

All patients presenting to the ED during this time period had a data form completed by registration personnel. Patients presenting in critical or unstable condition had as much information completed as possible, but not every survey item could always be completed because of the patient’s condition. Extensive demographic information was collected, including age, gender, and area of residence. Additional information was collected on patients with traumatic conditions including the mechanism of injury, place of injury, and body site of injury. Any type of injury was included as traumatic (e.g., lacerations, abrasions, strains, sprains). If a patient presented with a pain complaint (e.g. back pain), registration personnel were instructed to ask if the pain was caused by an injury. If answered affirmatively, the complaint was considered traumatic. Additionally, registration personnel asked injured patients if they were requesting a police report. A police report in Guyana is a legal document describing injuries sustained. It is done in accordance with the Evidence Act of the country and directed to the Magistrate handling a legal complaint [15]. Registration personnel also specifically asked injured patients at the time of presentation “Was the injury inflicted by a domestic partner?” At the time of the study, no referral procedures were in place for patients who disclosed IPV. Data were entered into an Excel spreadsheet.

This study is a retrospective analysis of the ED quality assurance database to determine the prevalence of IPV among patients presenting with acute traumatic injuries. No medical record review was performed. Patients 18 years or older presenting with acute traumatic conditions were included in the analysis. Patients without documentation in the database if the injury was a result of IPV were excluded from the analysis. Relative risks and 95% confidence intervals were calculated with Stata 10.1 (StataCorp LP, College Station, TX). GPHC provided written permission to use the quality assurance database, and Vanderbilt University’s Institutional Review Board approved the study as exempt.

## Results

During the 2- week study period, 598 adult patients with traumatic injuries presented to the GPHC ED. One hundred twenty- three patients (20.6%) did not have documented information about IPV on the data form and were excluded, leaving 475 patients for analysis. Overall, 38 (8%) of the 475 patients stated that a domestic partner inflicted their current injury. Thirty- one of the 38 (81.6%) patients reporting IPV were female. Of all injured females, 16% (31 of 194) stated that IPV was the cause of their injury. Comparatively, 2.5% (7 of 281) of all injured males stated that IPV was the cause of their injury (RR 6.4; 95% CI 2.9-14.3). Thirty- one of the 67 (46.3%) women presenting with assaults admitted IPV as the cause. The mean age for patients reporting IPV was 34.2 years (median 33 years), and the mean age for those not reporting IPV was 38.5 years (median 37 years).

The most common injuries were to the head, neck, or face (18 of 38, 47.4%) with extremity injuries the next most common (12 of 38, 31.6%). A blunt mechanism of injury was seen in 32 patients (84.2%), and a penetrating mechanism was seen in 6 (15.8%). Most (28 of 38, 73.7%) injuries occurred at the home of the patient. Almost all (37 of 38, 97.4%) of the patients reporting IPV resided in the administrative region (Region 4) surrounding the capital city of Georgetown. Most (37 of 38, 97.4%) came to the ED by bus, taxi, or private vehicle. One patient was transported by ambulance, and none were transported by police vehicle. One patient was admitted to the hospital for treatment of her injuries. A police report was requested at the time of presentation by 60.5% (23 of 38) of all patients reporting IPV and 67.7% (21 of 31) of women reporting IPV.

## Discussion

IPV occurs globally, but the rate of IPV differs markedly between societies. The World Health Organization (WHO) multi- country study on women’s health and domestic violence against women interviewed over 24,000 women at 15 sites in ten different countries and found a wide range in the lifetime prevalence. The rates ranged from 15% of Japanese women that had ever experienced physical or sexual violence by an intimate partner to 71% of provincial Ethiopian women [1]. Our study confirms a high prevalence of current IPV in women with acute traumatic injuries presenting to the GPHC ED in Guyana. It is difficult to compare the rates seen in this study with those seen in United States (US) emergency departments because of differences in study methodologies. One recent study from an urban US public hospital ED found a rate of 32.3% in women with non- verifiable injuries (i.e., assaults and falls), while 46.3% of our population with similar types of injuries admitted to IPV [16]. Overall, it is estimated that between 1–7% of women presenting to US emergency departments have acute injuries as a result of IPV, and 22% of women in the US will experience IPV at some point in their lifetime [5,17].

To adequately understand IPV, it must be viewed as more than abusive behavior. At the core, it is fueled by perceptions of gender inequality that permeate all aspects of society and extend beyond any one individual or family [18]. Gender inequality is woven into cultural norms [18]. Many women are even reluctant to disclose IPV because they themselves view the behavior as “normal” or “not serious” [1]. These women fear that they will not be believed, will not be helped, or will bring shame to their family [1]. Guyana is no exception. The Guyana National Policy on Domestic Violence as well as the Association of Caribbean Commissioners of Police acknowledge gender bias as a root cause of IPV and recognize the difficulty in changing these deeply entrenched, widespread views [10,19].

Although physical injuries may be the most obvious, the impact of IPV reaches further. In the World Health Organization multi- country study, women who had ever experienced IPV were significantly more likely to report poor or very poor health compared to matched controls that had never experienced IPV [1]. Chronic pain, central nervous system symptoms, gastrointestinal symptoms, and gynecological problems are all more common in victims of IPV [2]. Sexual abuse puts victims at risk for sexually transmitted infections, including HIV. Mental health is also negatively affected. Depression and post- traumatic stress disorder are common among IPV victims, and themselves carry a substantial risk of death and disability [2]. Clearly, recognizing and preventing acts of IPV would contribute to improved overall health and general well- being .

The healthcare system plays a prominent role in the diagnosis and management of IPV, as most victims will present for healthcare at some time [5]. Despite this, barriers exist within the healthcare setting that prevent IPV from being disclosed. In a survey of practicing physicians and nurses, inadequate preparation was identified as a key barrier to routine inquiry about IPV [14]. Formal training of healthcare professionals has been shown to increase knowledge and self- efficacy in discussing IPV as well as to improve attitudes about IPV [20-23]. This makes the establishment of healthcare provider education programs in Guyana a prime target for interventions to help combat IPV and is consistent with the needs assessment of the country [10].

IPV research has largely focused on women because the overwhelming majority of victims are women and women suffer more serious physical injuries [5]. However, men are also victimized, and there is a paucity of data on IPV among men. At the time of this study, screening tools for males had not been properly validated and the efficacy of ED screening and the true prevalence remain unknown [17,24]. The issue is further complicated by the fact that male IPV victims may also be perpetrators [17]. Our study found a prevalence rate of IPV among injured males of 2.5%. It is likely that social and cultural barriers prevent men from disclosing IPV, and more research is needed to determine the true extent of this problem.

Injuries to the head, neck, and face without a verifiable etiology are associated with a higher likelihood of IPV [16]. We also found that injuries to the head, neck, and face were common among IPV victims, occurring in almost one half of patients. Only one patient (2.6%) required admission to the hospital. While this low admission rate might seem surprising, in a study of US ED visits coded for IPV only 1% of IPV visits resulted in hospital admission [25]. Additionally, the more critically ill patients in our study might not have been asked about IPV because of the severity of their injuries.

In Guyana, a police report for judicial proceedings is required from a medical professional when the victim is intending to levy charges [15]. We found that about two- thirds of the women had a police report completed in the ED, suggesting that most had entered or were intending to enter the legal system. In comparison, a US Department of Justice survey found that 26.7% of US women who were physically assaulted by an intimate filed a police report, and the WHO multi- country survey found that most women who were physically assaulted told family or friends rather than formal agencies [1,5]. Given the legal requirement for a police report in Guyana, it is likely that some of the victims in our study presented to the ED in order to have the police report completed by a physician rather than for treatment of their injuries.

## Limitations

This study has several limitations. As is often the case, our findings are likely an underestimate of the true prevalence of IPV in this population. There is no gold standard to diagnose IPV and no single screening tool that identifies all victims [26]. Patients presenting with acute medical, chronic medical, or mental health conditions were not asked about IPV in this study as it was initially done for quality assurance purposes and those populations were not targeted. Non- injury- related ED visits are more common than injury- related ED visits among women experiencing IPV [8,25]. Since data were collected for a quality assurance database, patients were asked a single scripted question about IPV. The use of a single question as a screening tool for IPV has a sensitivity of 54.5-71.4% in the US ED population [27]. If additional screening tools were employed, it is likely more patients would have disclosed IPV. Likewise, patients were not always interviewed in a private setting and were interviewed by personnel not trained in IPV recognition and management. Thus, a significant number of IPV victims may have been missed.

Self-report was used to identify patients whose injuries were the result of IPV. Due to the retrospective methodology of this study, no additional chart reviews were performed, and no attempt was made to confirm the reports of IPV or to identify additional patients that may have subsequently disclosed IPV to providers. It is also possible our study missed patients presenting with more serious injuries. Patients lacking IPV data (20.6% of patients presenting with traumatic conditions) may have been sicker, precluding registration personnel from asking the question regarding IPV.

## Conclusions

This preliminary study demonstrates a high prevalence of IPV in adult patients presenting to the GPHC ED with acute traumatic injuries. The rate of IPV among women found in this report appears to be as high as, or higher than, that found in similar studies done in the US. While Guyana is a small developing country, it is likely that our findings are representative of similar settings in the Caribbean and South America. Given the well- documented detrimental impact of IPV on health and general well- being , focused efforts on healthcare provider education should remain a high priority in Guyana. Focus group interviews with IPV victims may provide valuable information to shape such programs, and additional research should seek to delineate the true scope of the problem as well as to evaluate the effectiveness of any interventions.

## Abbreviations

IPV: Intimate partner violence; ED: Emergency Department; GPHC: Georgetown Public Hospital Corporation; WHO: World Health Organization.

## Competing interests

The authors declare that we have no competing interests.

## Authors’ contributions

KPP participated in data analysis and interpretation and drafted the manuscript. SR participated in study design and data analysis. DA participated in study design and data acquisition. NR participated in study design and critically revised the manuscript. SL participated in study design and data analysis. SWW participated in study design, data analysis and interpretation, and critically revised the manuscript. All authors read and approved the final manuscript.
